# Addressing docking pose selection with structure-based deep learning: Recent advances, challenges and opportunities

**DOI:** 10.1016/j.csbj.2024.05.024

**Published:** 2024-05-18

**Authors:** Serena Vittorio, Filippo Lunghini, Pietro Morerio, Davide Gadioli, Sergio Orlandini, Paulo Silva, Jan Martinovic, Alessandro Pedretti, Domenico Bonanni, Alessio Del Bue, Gianluca Palermo, Giulio Vistoli, Andrea R. Beccari

**Affiliations:** a Dipartimento di Scienze Farmaceutiche, Università degli Studi di Milano, Via Luigi Mangiagalli 25, I-20133 Milano, Italy; b EXSCALATE, Dompé Farmaceutici SpA, Via Tommaso de Amicis 95, 80123 Naples, Italy; c Pattern Analysis and Computer Vision, Fondazione Istituto Italiano di Tecnologia, Via Morego, 30, 16163 Genova, Italy; d Dipartimento di Elettronica Informazione e Bioingegneria, Politecnico di Milano, Via Ponzio 34/5, I-20133 Milano, Italy; e SCAI, SuperComputing Applications and Innovation Department, CINECA, Via dei Tizii 6, Rome 00185, Italy; f IT4Innovations, VSB – Technical University of Ostrava, 17. listopadu 2172/15, 70800 Ostrava-Poruba, Czech Republic; g Department of Physical and Chemical Sciences, University of L′Aquila, via Vetoio, L′Aquila 67010, Italy

**Keywords:** Artificial intelligence, Deep learning, Molecular docking, Scoring functions, Pose selection

## Abstract

Molecular docking is a widely used technique in drug discovery to predict the binding mode of a given ligand to its target. However, the identification of the near-native binding pose in docking experiments still represents a challenging task as the scoring functions currently employed by docking programs are parametrized to predict the binding affinity, and, therefore, they often fail to correctly identify the ligand native binding conformation. Selecting the correct binding mode is crucial to obtaining meaningful results and to conveniently optimizing new hit compounds. Deep learning (DL) algorithms have been an area of a growing interest in this sense for their capability to extract the relevant information directly from the protein-ligand structure. Our review aims to present the recent advances regarding the development of DL-based pose selection approaches, discussing limitations and possible future directions. Moreover, a comparison between the performances of some classical scoring functions and DL-based methods concerning their ability to select the correct binding mode is reported. In this regard, two novel DL-based pose selectors developed by us are presented.

## Graphical Abstract

**Figure:**
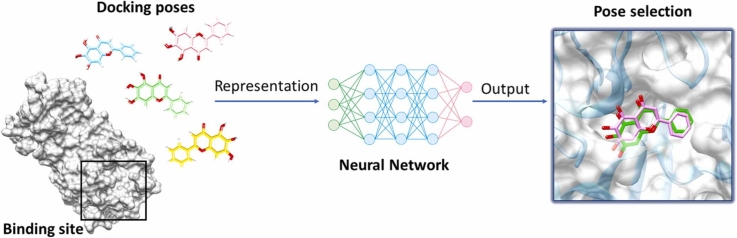

## 1 Introduction

Molecular docking is a well-established technique in structure-based drug design (SBDD).[1], [2], [3] The aim of such approach is not only to determine the binding conformation of a ligand within the target binding site, but also to estimate the binding affinity (BA) of the resulting complex.[4] For this purpose, two main steps are carried out in a typical docking calculation: sampling and scoring. The former explores different conformations of the ligand within the binding pocket, while the latter evaluates the generated docking poses by assigning a score which measures the complementarity between ligand and target. Docking scores are computed by appropriate mathematical or predictive models known as scoring functions (SFs).[5] Based on the computed scores, the most reliable binding pose is selected, and ligands are ranked against each other.[6] In a typical docking experiment, the top ranked compounds are considered to be the most promising hits for experimental evaluations; therefore, ligand scoring represents a crucial step as it strongly affects docking outcomes.[5].

The SFs employed by most of the docking tools are usually denoted as classical SFs including physical, empirical and knowledge-based SFs.[7] These SFs usually assume a predetermined functional form, normally a linear regression model, to describe the relationship between the descriptors used to characterize the complex and the binding affinity.[8] However, the linear relationship adopted by classical SFs is not valid for every complex leading to low accurate predictions.[9], [10] In recent decades, the implementation of artificial intelligence (AI) algorithms in drug design [11], [12], [13] enabled the development of AI-based SFs which are also able to capture non-linear relationships from data, allowing the predetermined linear regression models imposed by classical SFs to be overcome. Indeed, AI-based approaches learn the functional form directly from the data, thus capturing information about the ligand-protein interaction that is not easy to model explicitly.[14].

According to the Comparative Assessment of Scoring Functions 2016 (CASF-2016) benchmark, a reliable SF must accomplish four main tasks that can be assessed in terms of scoring, ranking, screening and docking power.[15] The scoring power describes the ability of the SF to compute a score that is linearly correlated with the experimentally measured BA value of solved protein-ligand complexes. The Person’s correlation coefficient is the metric used to estimate the correlation between the computed score and the experimental value. The ranking power refers to the capability to correctly rank a set of ligands bound to the same protein and is measured by the Sperman’s rank-correlation coefficient. Instead, the screening power is related to the ability to retrieve true binders among a set of random compounds, usually denoted as decoys, and it is expressed as an enrichment factor (EF) which measures the number of active molecules identified among the top *n* ranked compounds. Finally, the docking power represents the ability of the SF to select the native binding conformation of a ligand among a set of computer-generated poses.[16], [17] The Root Mean Square Deviation (RMSD) is usually employed to quantify the distance between the predicted and native poses. Generally, a RMSD value lower than 2 Å indicates a successful binding mode prediction.[18] An ideal SF should be able to rank the near native pose as the top one. Most of the SFs are trained to predict the BA and assume that the pose characterized by the highest affinity is with high probability the near native one.[19] However, the outcomes of CASF-2016 benchmark highlighted that there is no correlation between scoring and docking power.[15] BA-based SFs are trained using the single binding conformation of the ligand found in the experimental complex and, therefore, they are not conceived to specifically identify the native pose among the different conformers yielded by a typical docking calculation[20].

Selecting the true binding mode is crucial to obtaining meaningful scores, ranking and correctly discriminating between binders and non-binders.[21] Moreover, the identification of the correct target-ligand interactions plays a pivotal role for structure-activity relationship investigations and to rationally design new compounds for hit-to-lead and lead optimization purposes.[22].

Evaluating the BA of non-native poses is almost impossible; therefore, to improve pose selection in docking experiments, other strategies must be undertaken. In recent years, deep learning (DL) methods have gained increasingly more attention than traditional machine learning (ML) approaches due to their ability to automatically extract the relevant features directly from the protein-ligand structural representation without the need of building task-related specific features.[23] In this scenario, the present review aims to discuss the recent advances concerning the development of DL-based methods specifically tailored for the prediction of the near native ligand binding pose without considering the binding affinity in the training phase. Furthermore, a comparison concerning the ability of three traditional SFs, namely PLANTS ChemPLP, Glide XP and Autodock Vina and some selected DL-based approaches, including two methods developed by us, to correctly pick the near-native conformations among an array of docking poses is provided.

## 2 Deep learning: an overview

**Fig. 1:**
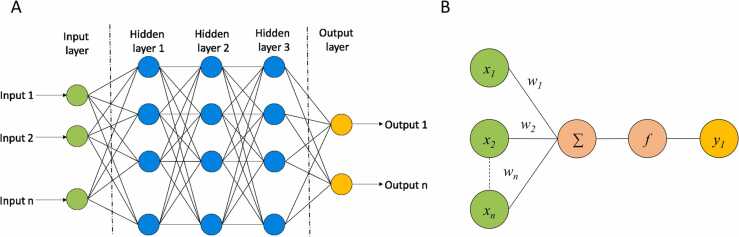
A) General architecture of a NN composed of one input layer (green), three hidden layers (cyan) and one output layer (yellow). B) Input processing at a node of a NN. Each hidden node takes the weighted sum of its inputs which is used to compute the output through a non-linear activation function.

DL methods derive from artificial neural networks (ANN), a ML approach inspired by the functional mechanisms of neurons in the brain. The simplest neural network (NN) is composed of an input layer, one or more hidden layers and an output layer (Fig. 1A). Similarly to neurons, an NN node receives different input signals and provides a response by computing a weighted sum of the inputs and the application of a nonlinear activation function. Then, the output signal is transmitted to the connected nodes, also called neurons (Fig. 1B).[24] In this way, the input variables are transformed through the hidden nodes until passing to the output layer. The latter processes the information received from the previously hidden layer and computes the output. Based on the type of NN, the nodes in neighboring layers can be fully or partially connected. The training of an NN consists of the iterative adjustment of the weight value in order to minimize the error between the predicted and the true value.[25] Differently from traditional ANNs, DL supports a greater number and variety of hidden layers which allow for learning multiple levels of representations thus being able to handle more complex problems.[26].

Multilayer perceptrons (MLP), or feed-forward neural networks, represent the most basic form of ANN and have been widely used in QSAR studies.[27], [28] In this type of architecture, the input is processed in one direction without the formation of loops or backwards connections between the nodes.[29].

Among the DL algorithms, convolutional neural networks (CNN), graph neural network (GNN) and, more recently, transformers have been largely exploited in SBDD, and in particular, in molecular docking.[30], [31], [32].

**Fig. 2:**
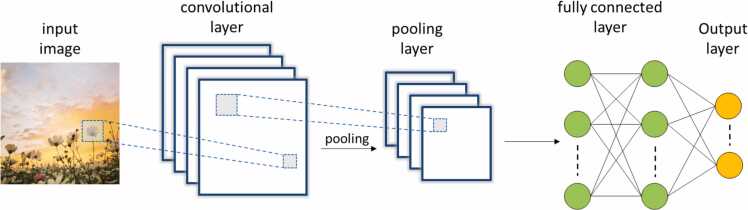
Architecture of a CNN.

A CNN is a type of NN used for processing data with a grid-like topology such as images or 3D voxels.[33] A CNN structure usually comprises three types of layers: convolutional, pooling and fully connected layers (Fig. 2). Convolutional and pooling layers are involved in feature extraction, while the fully connected layer integrates the extracted features and performs the classification or regression tasks.[34] In more details, a CNN uses as input a multidimensional array of numbers, called tensor, and performs feature extraction by executing an operation defined as convolution. Specifically, convolution consists of applying a smaller array of weights known as kernel on the input array and computing the dot product between each entry of the kernel and the receptive field of the input. The kernel slides across the tensor and the convolution is carried out at each position of the input yielding a 2D array of output values called feature map. Such process is repeated using multiple kernels that will extract different attributes of the input producing several feature maps that will be transferred to the pooling layer.[33] The latter is employed to reduce the dimensionality of the feature maps while preserving the most relevant information. Finally, the fully connected (FC) layer consists of different hidden layers in which each neuron is connected to all the neurons of the previous layer as in traditional ANNs. The FC layer elaborates the data derived from convolutional and pooling procedures computing the final output.[26], [34].

Considering the outstanding results obtained for image recognition, CNN has been proposed for SBDD due to its ability to extract the most relevant features directly from the 3D structure of the protein-ligand complex.[35] For this purpose, the complex coordinates are represented by 3D grids composed of voxels featurized with physicochemical descriptors defining the characteristics of the complex.[30], [32].

**Fig. 3:**
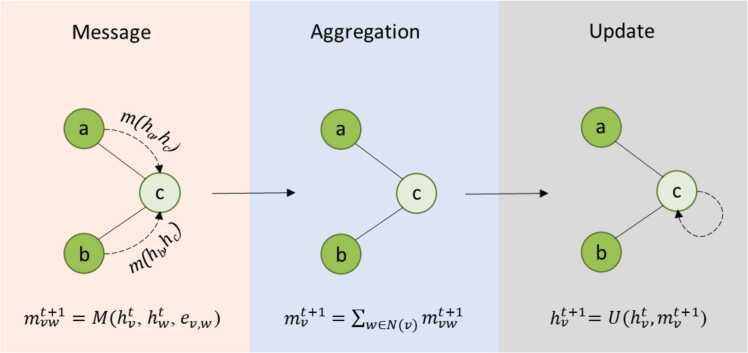
Schematic representation of message passing phases. Each node computes and passes a message to its neighbouring nodes. The incoming messages are aggregated and exploited to update the node features.

Another way to represent molecular structure is a graph consisting of a set of vertices, or nodes, representing the atoms, and edges corresponding to the bonds connecting the atoms.[36] This kind of representation is used as input for graph neural networks (GNNs), NN models based on a message passing scheme where each node updates its state by aggregating the data derived from the neighboring nodes.[37] Despite several GNN architectures having been developed,[38] most GNNs fall into the Message Passing Neural Network (MPNN) framework.[39] A typical MPNN consists of three phases: message, aggregation, and update (Fig. 3). In the message phase, each node *v* spreads the information to neighboring nodes using a message function *M(∙)*. These messages are then aggregated, typically through summation. Finally, each node updates its hidden state *h*_*v*_ using an update function *U(∙)* and exploiting the aggregated messages. This process repeats for a set number of iterations to capture graph structural information. A readout function *R(∙)* is then employed to aggregate all node vectors into a graph-level representation for prediction. [40], [41].

In a graph-based model, the most common features associated with the nodes representing the atoms include atom type, formal charge, hybridization, chirality, aromaticity and other atomic properties. Instead, the descriptors used for edges, representing the bonds, comprise bond type, conjugation, stereochemistry and ring (Table 1).[40] These features can be learned from 2D molecular graphs except for chirality which requires 3D spatial information. To overcome this limitation, 3D molecular graphs encoding the 3D arrangement of atoms can be used. Apart from chirality, the 3D molecular graph representation allows additional information to be included such as torsion angle and bond length enabling the discrimination between different isomers and conformers, thus yielding a more complete description of the molecular conformation which is crucial for SBDD purposes.[42].

**Table 1:** Attributes commonly associated to molecular graph nodes and edges.

Graph level	Feature	Description
node	atom type	type of atom (C, O, N etc.)
	charge	formal charge
	hybridization	sp, sp^2^, sp^3^ sp^3^d, or sp^3^d^2^
	aromaticity	if the atom is part of an aromatic system
	chirality	R or S
	n° bonds	number of covalent bonds involving the atom
	n° Hs	number of hydrogens bonded to the atom
edge	bond type	single, double, triple or aromatic
	conjugation	if the bond is conjugated
	stereochemistry	none, any, Z, E
	ring	if the bond is part of a ring

**Fig. 4:**
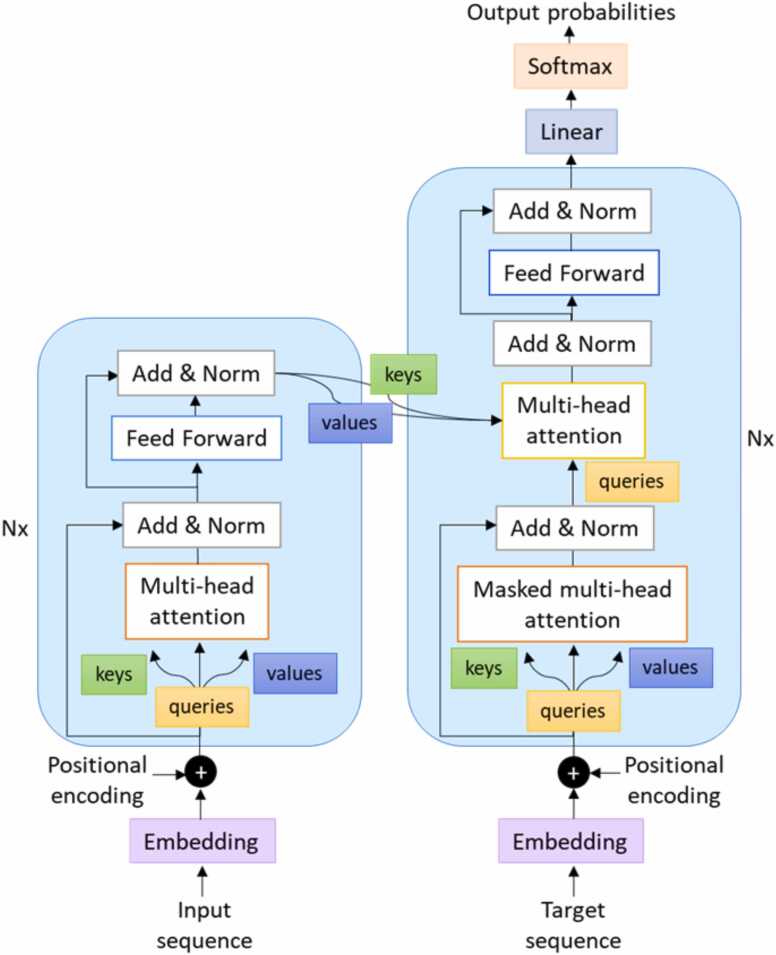
Transformer architecture.

Recently, transformer architectures have also been explored for drug design applications, including de novo design, reactions and molecular property prediction.[43], [44], [45], [46], [47] Transformers have been developed for sequence modelling such as natural language processing (NLP). This type of NN is based on the attention mechanism which enables the model to focus on the parts of the input that are crucial for the prediction by assigning different weights to each part.[48] The original structure of transformers was proposed by Vaswani *et al.* [49] and relies, at its core, on the so-called attention layers. Attention layers are responsible for computing attention scores and applying them to the input data. They play a crucial role in enabling the model to selectively focus on different parts of the input sequence. Each attention module usually contains multiple parallel attention heads and position-wise fully connected feed-forward network sub-layers (Fig. 4). In transformers, the process of embedding the input into a sequence of vectors is also enhanced by positional encoding vector representation, which captures the position of the vector in the sequence [51]. Differently from other NNs, such as recurrent Neural Networks (RNN), in which the input text is processed sequentially and the past information is preserved through the hidden states, transformers allow the input sequence to be processed as a whole, enabling parallel computation and alleviating the problem of long-term dependencies.[50].

## 3 Data availability

The performance of a DL model strongly relies on the quality and size of the training data. DL algorithms usually require large datasets for training models which yield accurate predictions. [51], [52] In Table 2 the datasets of experimental protein-ligand structures, which are frequently exploited for the development of structure-based DL models, are reported. Among them, the PDBbind database [53], [54] is the most used for molecular docking applications. In its current version, 2020 (accessed in April 2024), it collects 19443 protein-ligand complexes with the associated BA data. To provide high quality data, the PDBbind was split into “refined” and “general” sets. Originally, the former included complexes with a resolution lower than or equal to 2.5 Å, with a single ligand non-covalently bound to the target and containing common organic elements, namely C, N, O, P, S, F, Cl, Br, I and H. Furthermore, only complexes with known equilibrium constants, *K*d or *K*i, were included in the refined set. [54] In the following releases, complexes with missing residues or sidechains within 8 Å from the binding site or with significant steric clashes between ligand and protein heavy atoms were also discarded. [15].

**Table 2:** Databases used for the development and validation of structure-based DL models.

Database	n° of complexes	Source
PDBbind 2020	19,443	pdbbind.org.cn
CASF-2016	285	pdbbind.org.cn/casf.php
Binding MAOD 2020	41,409	bindingmoad.org
Astex diverse set	85	ccdc.cam.ac.uk/support-and-resources/downloads
CSAR-NCS HiQ	466	csardock.org

The CASF dataset was designed as a benchmark to evaluate the performances of SFs in terms of scoring, ranking, docking, and screening power. Originally developed to compare classical SFs, today it represents a standard also for the validation of AI-based SFs. Its latest release (2016) contains 285 protein-ligand complexes selected from the PDBbind refined set v. 2016 by applying the following rules. Proteins with a sequence similarity higher than 90% were clustered together and five representative structures were selected based on their BA values. In more details, the complexes endowed with the highest and the lowest BA values were selected along with other three complexes with BA values evenly distributed between the lowest and the highest BA. The electron density map of each complex was examined to evaluate the quality of the complex as well as ligand molecules to ensure that the dataset contains no identical ligands or stereoisomers. [15] The CASF dataset constitutes the PDBbind core set.

The Binding MOAD (Mother of All Databases) comprises high quality protein-ligand structures selected from the Protein Data Bank based on the following filters. Complexes with a resolution greater than 2.5 Å, NMR structures, theoretical models or covalently bound ligands were removed. Structures characterized by the presence of chains of i) four nucleotides or less, ii) ten amino acids or less, and iii) cofactors were kept. The primary literature references were checked for all the structures and affinity data expressed as *K*d or *K*i were considered. To avoid data redundancy, proteins were grouped according to their sequence similarity. For each cluster a representative entry was chosen based on various filters such as higher potency if the affinity data was available, the best resolution, the absence of mutations and the most recent deposition date. The latest release of Binding MOAD (2020) contains 41409 protein-ligand complexes and offers useful tools to evaluate the similarity of ligands and binding sites. [55], [56].

The Astex diverse set includes 85 biologically relevant protein-ligand complexes and is usually employed for the evaluation of SFs. In this case proteins were also clustered according to sequence similarity and ligand analysis was carried out to identify compounds of pharmacological and agrochemical interest. Any clashes between protein and ligand atoms as well as electron density maps were checked to identify the best quality structures. [57].

Another dataset initially developed to evaluate the performances of SFs is the CSAR-NCS HiQ (Community Structure Activity Resource) database which collects structural data from industry and academics laboratories. The CSAR-NCS HiQ dataset is subdivided into Set1 and Set2 containing 176 and 167 entries, respectively. Overall, the database includes 52 proteins with two or more structures and 191 proteins with a single structure. Protein-ligand complexes were initially selected from the Binding MOAD and integrated with data from PDBbind according to the quality of protein and ligand structures. [58], [59].

Despite several efforts to improve data quality and reduce data redundancy, the databases currently available still offer a low number of high-quality structures often biased towards certain protein families and/or ligands classes, leading to models with low generalizability. [60] To increase the number of training samples, data augmentation strategies have been explored which, in the case of pose selection methods, mainly consist of generating multiple docking poses for each protein-ligand complex. However, this approach slightly improves the performance of some DL models [61], [62] and, therefore, new methods need to be investigated.

## 4 DL-based pose selection methods

### 4.1 CNN-based approaches

The first description concerning the CNN application to pose selection was reported by Ragoza and colleagues in 2017.[35] The authors developed a 3D-CNN model for pose classification trained on the CSAR-NRC HiQ dataset along with the CSAR HiQ Update set,[63] and externally validated on the PDBbind 2013 core set.[54] Ligands were docked with smina[64] by employing AutoDock Vina SF.[65] The poses characterized by heavy-atoms RMSD values less than 2 Å from the native x-ray pose were labelled as positive examples while those with RMSD higher than 4 Å were labelled as negative examples. The docking poses with RMSD values between 2 and 4 Å were discarded. The 3D protein-ligand complex was discretized into a grid of 24 Å per side centered around the binding pocket setting a resolution of 0.5 Å. Each grid point carried the information related to the type of heavy atom at that point stored as density distribution. The ability of the model to discern between low and high RMSD poses was evaluated in terms of inter-target and intra-target ranking. In the first case, the CNN model outperformed Autodock Vina SF, while the opposite was observed in the intra-target ranking.[35].

In 2020, the same research group further extended this network to perform both pose classification and affinity prediction.[62] For this purpose, they employed the same input representation to develop several models composed of a series of 3D convolutional and pooling layers followed by two separate fully connected layers which output the pose score and the binding affinity. The PDBbind v2016 database was opportunely partitioned for models training and evaluation. Moreover, the authors created a new training set, named CrossDocked2020 set, collecting 22.5 million poses obtained by docking ligands into non-cognate receptor structures, thus giving a more realistic description of a docking simulation whose aim is to predict the binding mode of new ligands into a given structure rather than re-docking them to their native structure. Overall, the results highlighted an improvement of the performances in both inter-target and intra-target pose ranking as well as satisfactory pose selection ability on the cross-docked poses if compared to Autodock Vina SF.

Jiang *et al.* proposed a 3D-CNN model, termed MedusaNet, able to predict the probability of a docking pose to be a good binding pose.[66] The model architecture comprised six convolutional layers followed by three fully connected layers and is trained on a refined set of the PDBbind dataset. Given a protein-ligand complex, positive and negative poses were first generated by means of MedusaDock;[67] a pose was considered to be positive if the RMSD value respective to the native pose is less than 2 Å. The obtained poses were then evaluated by the CNN model. Input data are represented as a 4D tensor of which the first three dimensions define the spatial coordinates while the fourth dimension determines the atom type. The energy score computed by MedusaDock is included as a feature in MedusaNet in the second fully connected layer. The final output is a value between 0 and 1 which indicates the likelihood of a pose to be a good binding pose. The outcomes revealed an improvement of the AUC and average RMSD values if compared to MedusaDock score, highlighting the potential of 3D-CNN models to guide and improve conventional protein-ligand docking experiments. Furthermore, the integration of MedusaNet allowed the computational cost to be reduced as the search process stopped when *k* good poses are identified by the CNN model instead of iterating until the global minimum energy has converged. [66].

Differently from the above reported methods based on a classification task, Bao and colleagues built a regression model named DeepBSP, able to predict the RMSD value of a docking pose with respect to the native binding conformation.[68] To achieve this goal, the authors adopted a 3D-CNN architecture similar to that employed by *k*_DEEP_ tool [69] and trained their models on the complexes from the PDBbind general set v2019 using the native binding conformations of the ligands and a set of decoy poses generated by Autodock Vina program. The resulting models were evaluated on the CASF-2016 benchmark which contains its own set of decoy poses computed by other docking tools. When the native structure was included in the decoy pose set, DeepBSP ranked third in terms of docking power compared to the other SFs benchmarked in CASF-2016. In contrast, when only the docking poses are present in the decoy set, DeepBSP displayed the best performance among the other SFs.[68].

In 2022, Shim *et al.* reported two binary classifiers for pose selection based on a 3D-CNN and on an attention-based point cloud network (PCN), respectively.[70] In the last case, the 3D atomic structural data are represented as a point cloud by collecting information across all atom positions and features. Therefore, PCN does not require complex voxelization and is faster when compared to a 3D-CNN. Apart from the 3D atom representations, protein-ligand interaction features were also employed in the model training. In more details, both 3D-CNN and PCN models were trained i) with and without protein-ligand interactions features, ii) with and without affine transformation, iii) with both protein-ligand interaction features and affine transformation. The PDBbind 2019 refined database was employed as a training set and CASF2016 as a test set. Considering that the CASF2016 dataset includes protein families similar to those of the training set the authors used a dataset composed of ion channels to perform an unbiased external validation. A docking pose was considered to be correct if its RMSD value to the reference native structure was less than 2.5 Å, while it is incorrect if its RMSD is greater than 6.0 Å. The obtained models proved to be valid tools to filter out incorrect poses, thus improving the correlation between the experimental binding affinity and the Vina and Glide docking scores of the poses classified as correct. The application of affine transformation and the use of the interaction features generally led to more accurate models. Overall, the 3D-CNN classifiers exhibited better and more stable performances than the PCN ones which might be ascribable to a less accurate spatial representation of the PCN models. In the same year, Zhang and colleagues published a CNN classification model called DeepBindBC able to identify native-like protein-ligand complexes.[71] The model was trained on the PDBbind v2018 dataset and tested on CASF2013, CSAR-HiQ_NRC and Astex Diverse sets. Crystal structures were labelled as positives, while the negative samples were obtained by randomly crossdocking selected non-native ligands to proteins from the PDBbind dataset. Interestingly, instead of using a 4D input matrix describing the 3D coordinates and atom types, the authors represent the interface contacts as a 2D map processing the information as image-like data, thus facilitating the model training. The output of the model is a value between 0 and 1, where values closer to 1 indicate a high probability that a given structure is the native-like complex. DeepBindBC outperformed Autodock Vina SF on the three test sets and also another DL SF, Pafnucy [72] on three datasets selected from DUD.E.[73].

Despite the quite satisfactory outcomes achieved by CNN methods, such approaches are computationally expensive. Indeed, convolutions are also performed on empty regions of the grid as the voxels contain void spaces where no atoms are located. Moreover, 3D-CNNs lack rotational invariance and, therefore, data augmentation, by using different orientations of the input structure, is required in the training phase.[36].

### 4.2 Graph-based approaches

To overcome the pitfalls associated with the grid-like representation of 3D-CNN, graph-based methods, that are often translation and rotational invariant, were adopted.[30].

In 2019, Lim *et al*. proposed a GNN based model with a gated-augmented attention layer (GAT) for virtual screening and pose selection tasks.[23] The 3D structural information was described by two matrices: one accounting only for covalent bonds and the other one representing both covalent and non-covalent intermolecular interactions. Furthermore, the bond distance was exploited to estimate the strengths of the interactions. During the model training, the graph features of the separated protein and ligand are subtracted from those of the respective complex, allowing the network to capture the intermolecular interactions rather than just learning some ligand patterns. The model was trained and tested on the PDBbind v.2018 set for pose classification and on the DUD.E dataset for virtual screening. The docking poses were generated with Smina and labelled as positive if the RMSD value respective to the native pose was less than 2 Å or negative if the RMSD was greater than 4 Å. The developed GNN model outperformed conventional docking in terms of both docking and screening power. One year later, Morrone and co-workers developed a modular graph convolutional neural network (GCN) for virtual screening and binding mode prediction.[74] GCN is a class of GNNs applying convolution operations to graphs.[26] The model employed two graphs as input. The first one (L) describes ligand connectivity while the second graph (LP) represents the protein-ligand contacts. Each graph is fed to a CNN and the resulting representations are supplied to further layers to obtain the output in a separated (L, LP) or combined (L+LP) manner. The modular nature of the network allows evaluating the contribution of the sole ligand structure and of the protein-ligand contacts to the prediction. On this basis, the authors trained L, LP and L+LP networks; moreover, for the binding pose selection task, the rank (R) of the poses predicted by the docking software (i.e. Autodock Vina) was included as a feature. The DUD.E and PDBbind v.2017 datasets were used to train and test the model for virtual screening and binding pose classification, respectively. A RMSD value of 2 Å was used as a cut-off to discriminate between positive and negative poses. As expected, the LP and the LP+L networks outperformed the L network in binding mode evaluation emphasizing the importance of learning protein-ligand contacts to predict the correct binding mode among different decoys poses. Nonetheless, Autodock Vina SF showed the best results in terms of success rate respective to the L+LP network. When the rank of the pose was fed to the network, the resulting L+LP+R model showed an increment of the success rate of about 5% compared to Vina. Interestingly, the L+LP+R models outperformed Vina score when applied to cross-docked datasets revealing itself to be a promising tool for real docking applications, where ligands are docked into non-cognate receptors structures.[74].

The DL-based pose selection strategies described so far rely on the pose sampling algorithms implemented in conventional docking tools which might require several runs to yield a good binding pose. Then, the generated poses are evaluated by the DL-based model to identify the near-native one. To simplify the sampling step, Jiang *et al.* introduced a GNN-based approach, named MedusaGraph, able to directly predict the binding poses and select the near-native one.[75] MedusaGraph consisted of two GNNs. The first one predicts the most plausible binding pose from an initial docking pose, while the second one assesses if the predicted pose corresponds to the near-native one. Both networks are trained and tested on the PDBbind 2017 refined set. The initial complex is generated by MedusaDock and converted into a graph representation which is used as input for the first GNN. Graph nodes are divided into flexible nodes, which include ligand atoms, and fixed nodes comprising receptor atoms. The pose prediction GNN is a vertex regression model predicting the movement of each flexible atom. The output of this network is a moving vector encoding the motion along each axis. The final coordinates of each flexible node are obtained by summing the initial coordinates and the moving vector. This process is repeated iteratively yielding the final docking pose which is transformed into a graph and transferred to the pose selection network. The latter is a binary classification model able to assess the reliability of the final pose yielded by the pose prediction network. In the training phase, the poses were considered to be good, if the RMSD value respect to the x-ray pose was less than 2.5 Å. Conversely, if the RMSD value was greater than 2.5 Å the pose was labelled as bad. The MedusaDock energy score was included in the model training. The final output indicates the probability of a docking pose to be a good or bad pose. First, the Authors compared the goodness of the final poses predicted by MedusaGraph, without pose selection, with respect to other approaches including MedusaDock, Autodock Vina, the CNN-based methods MedusaNet and AtomNet, and the GNN-based model Graph-DTI. As results, the poses predicted by MedusaGraph displayed an average RMSD comparable to that of the other methods. However, when the pose selector was applied, the average RMSD decreased, outperforming the other approaches. Interestingly, the application of MedusaGraph to the poses generated by Autodock led to a reduction of the average RMSD when compared to the result obtained by using only Autodock SF, revealing that this method can be applied to other docking programs to enhance their performances. The external evaluation on CASF dataset highlighted the better performances achieved by MedusaGraph prediction and selection networks compared to Autodock and MedusaDock.

The binding of a ligand to its receptor is a dynamic process that induces conformational changes in both entities. Therefore, docking a ligand into a rigid non-cognate receptor structure might lead to inaccurate predictions. Ensemble docking approaches using different protein conformations are usually employed to overcome this limitation. However, traditional scoring methods cannot integrate such receptor variability [76]. On the contrary, DL methods are be able to acquire information about how distinct protein conformations affect the binding mode of native and non-native ligands. On this basis, Stafford and colleagues proposed AtomNet PoseRanker (ANPR), a GCN classifier for the identification of near-native poses from docking, which is trained on protein conformational ensembles.[22] To account for protein flexibility, low-energy conformations of the receptor were generated for each complex of the PDBbind 2019 dataset and collected into the FlexPDBbind set. Crossdocking experiments were also performed to make the model able to recognize valid non-cognate ligand poses in different experimental protein conformations. Docking poses were generated by means of the smina program and those characterized by RMSD values lower than 2.5 Å to the crystal pose were considered a “hit”, while those having RMSD values higher than 4 Å were labelled as a “miss”. Protein and ligand atoms were encoded separately neglecting their covalent structure. To assess the contribution of the protein-ligand interface in pose classification, different layer configurations were tested including an only ligand atoms set, an only receptor atoms set and both ligand and receptor atoms. The results revealed that accounting for protein-ligand interface increased the model performance compared to considering only ligand atoms. Moreover, better performances were observed for the model trained on cross-docked structures if compared to that trained on the FlexPDBbind set. This might be explained by considering that the used structures are experimental conformations of the targets able to accommodate distinct binding modes.

Méndez-Lucio and co-workers exploited statistical potentials, which are based on protein-ligand pairwise distance likelihood, for pose prediction developing a geometric DL approach named DeepDock.[77] The model learns a potential that is tailored for each complex from the experimental protein-ligand structures contained in the PDBbind V.2019 database. In this method, the molecular surface of the binding site is represented as a polygon mesh, while the ligand is encoded as a 2D molecular graph. Target and ligand inputs are processed by two separate GNNs for features extraction. The node features are then pair-wise concatenated to reproduce the contacts between the ligand and its receptor. Finally, the concatenated features are employed as input of a mixture density network (MDN), which learns the probability density distribution of the distance values between each ligand and receptor nodes. A statistical potential is obtained by summing up the negative log-likelihood values of all pairs. DeepDock was evaluated as SF using the CASF2016 dataset outperforming the other benchmarked SFs in terms of screening power, while it ranks fifth in terms of docking power showing a success rate not far from that of the best performing SF Autodock Vina. In addition, DeepDock can be combined with an optimization algorithm to predict the pose associated with the global minimum of the potential that corresponds with high probability to the experimental ligand binding conformation. Inspired by this work, Shen *et al.* developed RTMScore, a SF for pose prediction and VS tasks by exploiting a similar architecture of DeepDock.[78] The main differences between the two approaches concern the target representation and the feature extraction module. In RTMScore the protein is represented as 3D residue graphs thus overcoming the sensitivity to the rotation associated with the mesh graph representation as implemented in DeepDock. Features extraction from protein and ligand graphs is performed by two independent graph transformers. The method employs an MDN to derive residue-atom distance likelihood potential: the mechanism learns the probability density distribution of the distance between each ligand’s atom and every point on the surface of the binding site. This involves the integration of the negative log-likelihood of all ligand atom pairs, akin to constructing a statistical potential. RTMScore was evaluated on the CASF2016 dataset achieving a success rate of 93.4% and 97.3% with the exclusion or the inclusion of the crystal poses, respectively, thus outperforming the other tested SFs and DeepDock in terms of docking power. Moreover, RTMScore displayed better results compared to the other SFs in cross-docking experiments. RTM can be used to directly score and rank docking poses.

### 4.3 Miscellaneous methods

CNN and graph-based approaches represent the most used methods applied for the identification of the near native ligand binding mode. Over the years, other DL architectures have been employed to address this task. In 2022, Guo and colleagues exploited Vision Transformer (ViT) to develop a SF named ViTRMSE able to recognize the near native pose among a pool of decoys poses.[79] ViT applies the transformers architecture for image classification. For this purpose, the image is represented as a sequence of patches that can be defined as small rectangular regions of the image. The generated patches are linearly embedded, position embeddings are added, and the resulting vectors sequence is given as input to the transformer. The self-attention mechanism allows the model to weigh the importance of the different image parts and to capture the long-range dependencies between the patches.[80] ViTRMSE employs a voxelized representation of the binding site that is then divided into patches and fed to the transformer architecture. The model was trained on the PDBbind V2019 database and tested on the CASF2016. The outcomes revealed that ViTRMSE showed a docking power superior to that of the CNN-based model DeepBSP and other SFs benchmarked in CASF2016.[79].

Wang *et al.* proposed a MLP model called DeepRMSD to predict the RMSD value between the docking pose and the native conformation.[19] The electrostatic and van der Waals interactions were used as features to characterize the protein-ligand complexes of the PDBbind V2019 databased used for the model training. When applied to the CASF2016 dataset, DeepRMSD yielded satisfactory performances in predicting the RMSD values of the docking poses characterized by RMSD values greater than 4 Å, while Autodock Vina SF displayed a better correlation in the RMSD range lower than 3 Å. To improve the overall performance, the authors combined DeepRMSD with Autodock Vina SF obtaining a new hybrid SF, named DeepRMSD+Vina, which outperformed both DeepRMSD and Autodock Vina SF in the low RMSD range. DeepRMSD+Vina performed well on the CASF2016 in terms of success rate, ranking second among the tested SFs with the first being RTMScore. Moreover, a gradient descent algorithm was implemented to optimize the quality of the docking poses. The outcomes highlighted that the optimization framework provided a high success rate with poses characterized by a low initial RMSD value. This can be explained by the fact that high RMSD poses require more consistent translational and rotational motions that can be constrained by the shape of the binding pocket. DeepRMSD+Vina outperformed both Autodock Vina SF and DeepBSP in crossdocking experiments performed both with and without pose optimization.

## 5 Comparison between classical SFs and DL-based pose selection methods

The last part of this review aims to provide a straightforward comparison between classical SFs and DL-based pose selectors concerning their ability to find the near native poses among a pool of decoys poses. For this purpose, a set of protein-ligand complexes was extracted from the PDBbind 2020 dataset as described in Supplementary Information. Three widely used docking tools, Glide, PLANTS and Autodock Vina were employed to generate the docking poses that were scored by i) the primary SF as implemented in each software,ii) two DL-based approaches published in literature, namely DeepDock[77] and RTM[78] and iii) two pose selectors specially developed by us by exploiting two already known DL architectures, GraphBAR[61] and DimeNet.[81] As discussed in the graph-based methods section, DeepDock and RTM share similar architectures. RTM was selected not only for its graph-based architecture but also for its interesting output layer mechanism based on Mixture Density Networks (MDN) [82]. Such a layer is trained to enforce high probability on exact geometries and consequently low probabilities elsewhere, making it suitable for ranking poses based on their likelihood. GraphBAR is a relatively simple GCN model originally developed for protein-ligand binding affinity prediction which we retrained for the different task of RMSD prediction (RMSD is calculated for each docked pose versus the native pose). In this approach, both protein and ligand are encoded as graphs, where each node (atom) carries 13 features including atomic number, hybridization and partial charge. While graph convolutions and gathering layers are quite standard, the GraphBAR pipeline introduces a novel mechanism which considers interactions at different scales. This relies on multiple parallel layers based on different adjacency matrices which encode atomic relationships at different distances. [61] DimeNet is a GNN approach conceived for the prediction of molecular properties and molecular dynamics. With respect to older approaches, such as GraphBAR, that represent molecules as graphs based solely on the distances between atoms, DimeNet introduces the interesting property of taking into account angles within atom triplets in its message passing algorithm. This innovative approach involves embedding messages exchanged between atoms instead of the atoms themselves, with each message associated with a specific direction in coordinate space. Notably, these directional message embeddings exhibit rotational equivariance, adjusting their associated directions as the molecule rotates. The message passing scheme employed in DimeNet is analogous to belief propagation, utilizing directional information by transforming messages based on the angles between them. The model leverages spherical Bessel functions and spherical harmonics to construct theoretically well-founded, orthogonal representations, thus surpassing the performance of prevalent Gaussian radial basis representations with significantly fewer parameters. [81] As for GraphBAR, DimeNet was retrained for the prediction of the RMSD value between a given computed pose and the respective native ligand binding conformation. Further details about the retraining of these models can be found in Supplementary Information.

**Fig. 5:**
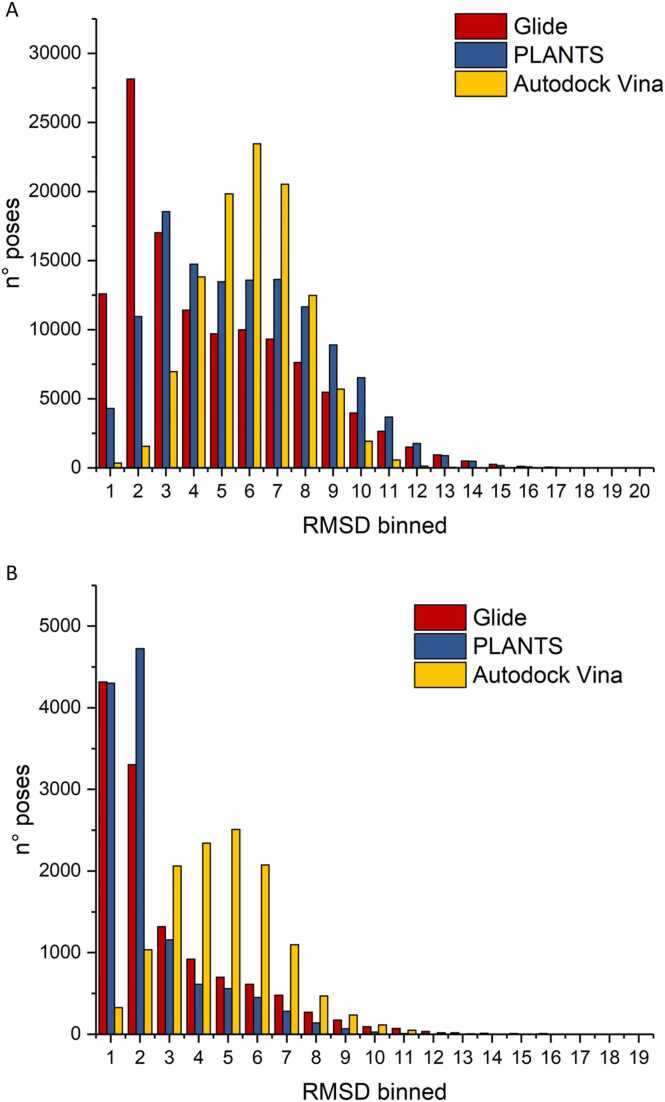
A) RMSD distribution of all the poses computed by each software. B) Distribution of the lowest RMSD values obtained for each complex.

Firstly, we analyzed the RMSD distribution of the poses computed by each software. As shown in Fig. 5A, Glide provided the highest number of poses with RMSDs lower than 2 Å (40728 out of 121240 poses), followed by PLANTS (15262 out of 123350) and lastly by Autodock Vina (1894 out of 107343). Notwithstanding these results, it is noteworthy that PLANTS was able to generate at least one pose with RMSD lower than 2 Å for the 73% of the complexes, while this was obtained for about the 62% and only for the 11% of the complexes by Glide and Vina, respectively (Fig. 5B). On this basis, PLANTS appears to be characterized by the best sampling ability among the three tools, at least in terms of self-docking.

To compare the performances of the selected methods in terms of pose selection, the mean RMSD value of the top-ranked poses predicted by each of the tested approaches was computed (Table 3). Among the classical SFs the best result was achieved by PLANTS ChemPLP with a mean RMSD value of 3.3 Å, while Vina performed the worst with a mean RMSD value of 5.2 Å. This outcome was quite expected as most of the complexes produced by Autodock Vina lack docking solutions close to the crystallographic conformation. Concerning the DL-based methods, DeepDock was the only DL- based approach able to outperform all the tested classical SFs reducing the mean RMSD value of more than 10% when analyzing the PLANTS and Glide poses and of about 4% in the case of Autodock Vina.

**Table 3:** Mean RMSDs computed considering the top-ranked docking solutions predicted by each approach. All analyses involved a common set of 12,235 poses.

SW	SF	Mean RMSD (Å)	Std.Dev.
Autodock Vina	Vina	5.2	2.2
Autodock Vina	DimeNet	5.1	2.1
Autodock Vina	DeepDock	5.0	2.2
Autodock Vina	GraphBAR	5.7	1.9
Autodock Vina	RTM_1	5.1	2.1
Autodock Vina	RTM_2	5.1	2.1
Autodock Vina	RTM_3	5.1	2.1
Autodock Vina	RTM Consensus	5.1	2.1
GLIDE	GLIDE XP	3.6	3.1
GLIDE	DimeNet	3.7	3.0
GLIDE	DeepDock	3.2	2.9
GLIDE	GraphBAR	4.2	3.1
GLIDE	RTM_1	3.5	3.0
GLIDE	RTM_2	3.5	3.1
GLIDE	RTM_3	3.5	3.1
GLIDE	RTM Consensus	3.5	3.1
PLANTS	PLANTS ChemPLP	3.3	3.1
PLANTS	DimeNet	3.4	2.9
PLANTS	GraphBAR	5.4	2.8
PLANTS	DeepDock	3.0	2.8
PLANTS	RTM_1	3.6	3.0
PLANTS	RTM_2	3.6	3.0
PLANTS	RTM_3	3.6	3.1
PLANTS	RTM Consensus	3.4	3.0

The application of DimeNet to Glide and PLANTS poses did not lead to any improvement with respect to the corresponding primary SFs, providing mean RMSD values comparable to those of ChemPLP and GLIDE XP. In contrast, a slight decrease of the mean RSMD value was observed when DimeNet was applied to Autodock Vina poses thus showing superior performances if compared to Vina SF. Instead, GraphBAR revealed itself to be dramatically worse than classical SFs yielding RMSD means greater than 4 Å. Regarding RTM, when applied to GLIDE and Autodock Vina poses, the obtained performances were slightly better than the respective primary SFs, while no improvements where registered when scoring PLANTS poses if compared to ChemPLP.

As expected, GraphBAR underperforms due to its outdated design, which does not take angles into account. On the contrary, DimeNet shows good results indicating the importance of considering angular interactions when modeling ligand protein interactions. RTM also exhibits favorable performance, highlighting the potential of a scoring function based on likelihood estimation.

DeepDock leverages geometric deep learning to create a protein-specific potential for ligand binding conformations to outperform other scoring approaches and tools. By training a deep neural network on a mixture model that considers ligand atom-surface point distances, DeepDock captures complex interactions, leading to enhanced accuracy in scoring molecular complexes over other approaches.

## 6 Conclusions and future outlooks

In this review we described the latest advancements concerning the development of pose selection strategies by exploiting DL algorithms. In addition, two novel pose selectors generated by us by using two already published architectures, DimeNet and GraphBAR, were proposed and their performances were compared to three classical SFs (Glide XP, PLANTS ChemPLP and Autodock Vina) and two known DL-based approaches, namely DeepDock and RTMscore. In our results, we observed that the ability of DL-based pose selection methods to pick the near native pose is comparable to that of classical SFs, except for DeepDock which yielded the best results among the tested approaches. Overall, our analysis, along with the recent findings presented in this review, revealed that the improvements offered by DL methods in docking pose selection are still moderate, highlighting some challenges associated with the use of DL in molecular docking.

The first issue is represented by the limited amount of training data. Indeed, the number of high-quality 3D protein-ligand structures in the available databases is restricted and often biased as for certain protein families only similar co-crystallized ligands are present leading to data redundancy. To partially solve this problem, data splitting based on structural similarity has been performed yielding models with lower performances but higher transferability. [83] In addition, molecules endowed with a low affinity are often underreported resulting in models with low generalizability. Data augmentation is often exploited in DL studies with limited training data; however, how to perform data augmentation in this case is far from being clear as simply generating various poses for the same protein-ligand complex proved ineffective.

Conformational flexibility plays a crucial role in SBDD. Despite some attempts to introduce target flexibility into DL models as in AtomNet PoseRanker [22], this aspect remains underexplored due to the scarcity of open-source datasets of conformational ensembles of protein-ligand complexes and the difficulty in selecting biologically relevant conformations of the target. Considering that the binding of a ligand to its receptor is a dynamic event, accounting for protein flexibility could positively influence the applicability of DL-based pose selection approaches.

Another important aspect to consider is that pose selection methods strongly rely on the conformational sampling algorithm adopted by conventional docking software. In this regard, the implementation of an NN able to predict the movement of ligand atoms within the protein binding site as in MedusaGraph [75] or of an optimization algorithm as in DeepDock [77], might help to overcome such limitation. It is worth mentioning that binding pose prediction without any prior conformational search, which is out of the scope of this review, has been addressed by generative models. [84], [85].

In general, DL models are characterized by poor interpretability and explainability. For this reason, they have been defined as “black boxes” due to the difficulty of understanding how the final decision is predicted. Attention mechanism has been identified as a promising solution to this problem as it allows identifying how different parts of the input influence the output. [86].

In conclusion, DL approaches appear to have the potential to tackle docking pose selection although several limitations need to be overcome. The availability of new experimental structural data along with the development of more explainable models could help to address the current challenges associated with the use of DL in SBDD. In addition, future research efforts should be focused on the experimental validation of these methods to fully understand the real applicability of DL in drug discovery projects.

## Declaration of Competing Interest

None.

## Footnotes

Appendix A Supplementary data associated with this article can be found in the online version at doi:10.1016/j.csbj.2024.05.024.

## References

[1] Vittorio S., Lunghini F., Pedretti A., Vistoli G., Beccari A.R. (2023). Ensemble of structure and ligand-based classification models for hERG liability profiling. Front Pharm.

[2] Meng X.-Y., Zhang H.-X., Mezei M., Cui M. (2011). Molecular docking: a powerful approach for structure-based drug discovery. Curr Comput Aided-Drug Des.

[3] Batool M., Ahmad B., Choi S. (2019). A structure-based drug discovery paradigm. Int J Mol Sci.

[4] Lin X., Li X., Lin X. (2020). A review on applications of computational methods in drug screening and design. Molecules.

[5] Ashtawy H.M., Mahapatra N.R. (2015). Machine-learning scoring functions for identifying native poses of ligands docked to known and novel proteins. BMC Bioinforma.

[6] Dhakal A., McKay C., Tanner J.J., Cheng J. (2022). Artificial intelligence in the prediction of protein-ligand interactions: recent advances and future directions. Brief Bioinform.

[7] Crampon K., Giorkallos A., Deldossi M., Baud S., Steffenel L.A. (2022). Machine-learning methods for ligand–protein molecular docking. Drug Discov Today.

[8] Li H., Peng J., Sidorov P., Leung Y., Leung K.S., Wong M.H. (2019). Classical scoring functions for docking are unable to exploit large volumes of structural and interaction data. Bioinformatics.

[9] Xiong G.L., Ye W.L., Shen C., Lu A.P., Hou T.J., Cao D.S. (2021). Improving structure-based virtual screening performance via learning from scoring function components. Brief Bioinform.

[10] Wójcikowski M., Ballester P.J., Siedlecki P. (2017). Performance of machine-learning scoring functions in structure-based virtual screening. Sci Rep.

[11] Shiota K., Suma A., Ogawa H., Yamaguchi T., Iida A., Hata T. (2023). AQDnet: deep neural network for protein–ligand docking simulation. ACS Omega.

[12] Jukič M., Bren U. (2022). Machine learning in antibacterial drug design. Front Pharm.

[13] Broz M., Jukič M., Bren U. (2023). Naive prediction of protein backbone phi and psi dihedral angles using deep learning. Molecules.

[14] Li H., Sze K.H., Lu G., Ballester P.J. (2021). Machine-learning scoring functions for structure-based virtual screening. Wiley Inter Rev Comput Mol Sci.

[15] Su M., Yang Q., Du Y., Feng G., Liu Z., Li Y. (2019). Comparative assessment of scoring functions: The CASF-2016 Update. J Chem Inf Model.

[16] Moon S., Zhung W., Yang S., Lim J., Kim W.Y. (2022). PIGNet: a physics-informed deep learning model toward generalized drug-target interaction predictions. Chem Sci.

[17] Li Y., Liu Z., Li J., Han L., Liu J., Zhao Z. (2014). Comparative assessment of scoring functions on an updated benchmark: 1. compilation of the test set. J Chem Inf Model.

[18] Bell E.W., Zhang Y. (2019). DockRMSD: An open-source tool for atom mapping and RMSD calculation of symmetric molecules through graph isomorphism. J Chemin-.

[19] Wang Z., Zheng L., Wang S., Lin M., Wang Z., Kong A.W.K. (2023). A fully differentiable ligand pose optimization framework guided by deep learning and a traditional scoring function. Brief Bioinform.

[20] Ashtawy H.M., Mahapatra N.R. (2018). Boosted neural networks scoring functions for accurate ligand docking and ranking. J Bioinform Comput Biol.

[21] Scardino V., Bollini M., Cavasotto C.N. (2021). Combination of pose and rank consensus in docking-based virtual screening: the best of both worlds. RSC Adv.

[22] Stafford K.A., Anderson B.M., Sorenson J., Van Den Bedem H. (2022). AtomNet poseranker: enriching ligand pose quality for dynamic proteins in virtual high-throughput screens. J Chem Inf Model.

[23] Lim J., Ryu S., Park K., Choe Y.J., Ham J., Kim W.Y. (2019). Predicting drug-target interaction using a novel graph neural network with 3d structure-embedded graph representation. J Chem Inf Model.

[24] Batool M., Ahmad B., Choi S. (2019). A structure-based drug discovery paradigm. Int J Mol Sci.

[25] Ekins S. (2016). The next era: deep learning in pharmaceutical research. Pharm Res.

[26] Chen H., Engkvist O., Wang Y., Olivecrona M., Blaschke T. (2018). The rise of deep learning in drug discovery. Drug Discov Today.

[27] Baskin I.I., Winkler D., Tetko I.V. (2016). A renaissance of neural networks in drug discovery. Expert Opin Drug Discov.

[28] Kleandrova V.V., Scotti L., Bezerra Mendonça Junior F.J., Muratov E., Scotti M.T., Speck-Planche A. (2021). QSAR modeling for multi-target drug discovery: designing simultaneous inhibitors of proteins in diverse pathogenic parasites. Front Chem.

[29] Yang X., Wang Y., Byrne R., Schneider G., Yang S. (2019). Concepts of Artificial Intelligence for Computer-Assisted Drug Discovery. Chem Rev.

[30] Isert C., Atz K., Schneider G. (2023). Structure-based drug design with geometric deep learning. Curr Opin Struct Biol.

[31] Krishnan S.R., Bung N., Vangala S.R., Srinivasan R., Bulusu G., Roy A. (2022). De novo structure-based drug design using deep learning. J Chem Inf Model.

[32] Özçelik R., van Tilborg D., Jiménez-Luna J., Grisoni F. Structure-based drug discovery with deep learning 2022. 10.1002/cbic.202200776. 37014633

[33] Yamashita R., Nishio M., Do R.K.G., Togashi K. (2018). Convolutional neural networks: an overview and application in radiology. Insights Imaging.

[34] Chen L., Li S., Bai Q., Yang J., Jiang S., Miao Y. (2021). Review of image classification algorithms based on convolutional neural networks. Remote Sens.

[35] Ragoza M., Hochuli J., Idrobo E., Sunseri J., Koes D.R. (2017). Protein-ligand scoring with convolutional neural networks. J Chem Inf Model.

[36] Meli R., Morris G.M., Biggin P.C. (2022). Scoring functions for protein-ligand binding affinity prediction using structure-based deep learning: a review. Front Bioinforma.

[37] Alon U., Yahav E. (2020). Bottle Graph Neural Netw its Pract Implic.

[38] Zhou J., Cui G., Hu S., Zhang Z., Yang C., Liu Z. (2020). Graph neural networks: a review of methods and applications. AI Open.

[39] Gilmer J., Schoenholz S.S., Riley P.F., Vinyals O., Dahl G.E. (2017). Neural Message Passing Quantum Chem.

[40] Reiser P., Neubert M., Eberhard A., Torresi L., Zhou C., Shao C. (2022). Graph neural networks for materials science and chemistry. Commun Mater.

[41] Buterez D., Janet J.P., Kiddle S.J., Oglic D., Liò P. (2022). Graph Neural Netw Adapt Readouts.

[42] Guo Z., Nan B., Tian Y., Wiest O., Zhang C., Chawla N.V. (2022). Graph-Based Mol Represent Learn.

[43] Zheng S., Rao J., Zhang Z., Xu J., Yang Y. (2020). Predicting retrosynthetic reactions using self-corrected transformer neural networks. J Chem Inf Model.

[44] Broberg J., Bånkestad M., Ylipää E. (2022). Pre-Train Transform Mol Prop Predict Using React Predict.

[45] Grechishnikova D. (2021). Transformer neural network for protein-specific de novo drug generation as a machine translation problem. Sci Rep.

[46] Morris P., St. Clair R., Hahn W.E., Barenholtz E. (2020). Predicting binding from screening assays with transformer network embeddings. J Chem Inf Model.

[47] Qian H., Lin C., Zhao D., Tu S., Xu L. (2022). AlphaDrug: protein target specific de novo molecular generation. PNAS Nexus.

[48] Tang Y. (2023). Deep learning in drug discovery: applications and limitations. Front Comput Intell Syst.

[49] Vaswani A., Shazeer N., Parmar N., Uszkoreit J., Jones L., Gomez A.N. (2017). Attention is all you need. Adv Neural Inf Process Syst.

[50] Zhang S., Fan R., Liu Y., Chen S., Liu Q., Zeng W. (2023). Applications of transformer-based language models in bioinformatics: a survey. Bioinforma Adv.

[51] Taye M.M. (2023). Understanding of machine learning with deep learning: architectures, workflow, applications and future directions. Computers.

[52] Sarker I.H. (2021). Deep learning: a comprehensive overview on techniques, taxonomy, applications and research directions. SN Comput Sci.

[53] Wang R., Fang X., Lu Y., Wang S. (2004). The PDBbind database: collection of binding affinities for protein−ligand complexes with known three-dimensional structures. J Med Chem.

[54] Wang R., Fang X., Lu Y., Yang C.-Y., Wang S. (2005). The PDBbind database: methodologies and updates. J Med Chem.

[55] Hu L., Benson M.L., Smith R.D., Lerner M.G., Carlson H.A. (2005). Binding MOAD (Mother of All Databases). Proteins Struct Funct Genet.

[56] Smith R.D., Clark J.J., Ahmed A., Orban Z.J., Dunbar J.B., Carlson H.A. (2019). Updates to Binding MOAD (Mother of All Databases): polypharmacology tools and their utility in drug repurposing. J Mol Biol.

[57] Hartshorn M.J., Verdonk M.L., Chessari G., Brewerton S.C., Mooij W.T.M., Mortenson P.N. (2007). Diverse, high-quality test set for the validation of protein-ligand docking performance. J Med Chem.

[58] Dunbar J.B., Smith R.D., Yang C.Y., Ung P.M.U., Lexa K.W., Khazanov N.A. (2011). CSAR benchmark exercise of 2010: selection of the protein-ligand complexes. J Chem Inf Model.

[59] Dunbar J.B., Smith R.D., Damm-Ganamet K.L., Ahmed A., Esposito E.X., Delproposto J. (2013). CSAR data set release 2012: ligands, affinities, complexes, and docking decoys. J Chem Inf Model.

[60] Özçelik R., van Tilborg D., Jiménez-Luna J., Grisoni F. (2023). Structure-based drug discovery with deep learning*. ChemBioChem.

[61] Son J., Kim D. (2021). Development of a graph convolutional neural network model for efficient prediction of protein-ligand binding affinities. PLoS One.

[62] Francoeur P.G., Masuda T., Sunseri J., Jia A., Iovanisci R.B., Snyder I. (2020). Three-dimensional convolutional neural networks and a crossdocked data set for structure-based drug design. J Chem Inf Model.

[63] Dunbar J.B., Smith R.D., Yang C.-Y., Ung P.M.-U., Lexa K.W., Khazanov N.A. (2011). CSAR Benchmark Exercise of 2010: Selection of the Protein–Ligand Complexes. J Chem Inf Model.

[64] Koes D.R., Baumgartner M.P., Camacho C.J. (2013). Lessons Learned in Empirical Scoring with smina from the CSAR 2011 Benchmarking Exercise. J Chem Inf Model.

[65] Trott O., Olson A.J. (2010). AutoDock Vina: Improving the speed and accuracy of docking with a new scoring function, efficient optimization, and multithreading. J Comput Chem.

[66] Jiang H., Fan M., Wang J., Sarma A., Mohanty S., Dokholyan N.V. (2020). Guiding conventional protein-ligand docking software with convolutional neural networks. J Chem Inf Model.

[67] Wang J., Dokholyan N.V. (2019). MedusaDock 2.0: efficient and accurate protein–ligand docking with constraints. J Chem Inf Model.

[68] Bao J., He X., Zhang J.Z.H. (2021). DeepBSP-a machine learning method for accurate prediction of protein-ligand docking structures. J Chem Inf Model.

[69] Jiménez J., Škalič M., Martínez-Rosell G., De Fabritiis G. (2018). KDEEP: protein-ligand absolute binding affinity prediction via 3D-convolutional neural networks. J Chem Inf Model.

[70] Shim H., Kim H., Allen J.E., Wulff H. (2021). Pose classification using three-dimensional atomic structure-based neural networks applied to ion channel-ligand docking. J Chem Inf Model.

[71] Zhang H., Zhang T., Saravanan K.M., Liao L., Wu H., Zhang H. (2022). DeepBindBC: a practical deep learning method for identifying native-like protein-ligand complexes in virtual screening. Methods.

[72] Stepniewska-Dziubinska M.M., Zielenkiewicz P., Siedlecki P. (2018). Development and evaluation of a deep learning model for protein–ligand binding affinity prediction. Bioinformatics.

[73] Mysinger M.M., Carchia M., Irwin J.J., Shoichet B.K. (2012). Directory of useful decoys, enhanced (DUD-E): better ligands and decoys for better benchmarking. J Med Chem.

[74] Morrone J.A., Weber J.K., Huynh T., Luo H., Cornell W.D. (2020). Combining docking pose rank and structure with deep learning improves protein−ligand binding mode prediction over a baseline docking approach. J Chem Inf Model.

[75] Jiang H., Wang J., Cong W., Huang Y., Ramezani M., Sarma A. (2022). Predicting protein-ligand docking structure with graph neural network. J Chem Inf Model.

[76] Guedes I.A., Pereira F.S.S., Dardenne L.E. (2018). Empirical scoring functions for structure-based virtual screening: applications, critical aspects, and challenges. Front Pharm.

[77] Méndez-Lucio O., Ahmad M., del Rio-Chanona E.A., Wegner J.K. (2021). A geometric deep learning approach to predict binding conformations of bioactive molecules. Nat Mach Intell.

[78] Shen C., Zhang X., Deng Y., Gao J., Wang D., Xu L. (2022). Boosting Protein-Ligand Binding Pose Prediction and Virtual Screening Based on Residue-Atom Distance Likelihood Potential and Graph Transformer. J Med Chem.

[79] Guo L., Wang J. ViTRMSE: a three-dimensional RMSE scoring method for protein-ligand docking models based on Vision Transformer. Proc - 2022 IEEE Int Conf Bioinforma Biomed BIBM 2022 2022:328–33. 10.1109/BIBM55620.2022.9995694.

[80] Dosovitskiy A., Beyer L., Kolesnikov A., Weissenborn D., Zhai X., Unterthiner T. (2021). an Image Is Worth 16×16 Words: Transformers for Image Recognition At Scale. ICLR 2021 - 9th Int Conf Learn Represent.

[81] Gasteiger J., Groß J., Günnemann S. (2020). Directional message passing for molecular graphs. 8th Int Conf Learn Represent ICLR.

[82] Bishop C.M. (1994). Aston university. Mixture Density Netw.

[83] Yang J., Shen C., Huang N. (2020). Predicting or Pretending: Artificial Intelligence for Protein-Ligand Interactions Lack of Sufficiently Large and Unbiased Datasets. Front Pharm.

[84] Corso G., Stärk H., Jing B., Barzilay R., Jaakkola T. (2022). DiffDock: diffusion steps, twists. Turns Mol Docking.

[85] Masters M.R., Mahmoud A.H., Wei Y., Lill M.A. (2023). Deep learning model for efficient protein–ligand docking with implicit side-chain flexibility. J Chem Inf Model.

[86] Li A., Xiao F., Zhang C., Fan C. (2021). Attention-based interpretable neural network for building cooling load prediction. Appl Energy.

