# Supplementary material for: Addressing docking pose selection with structure-based deep learning: Recent advances, challenges and opportunities
Source: Comput Struct Biotechnol J. 2024 May 18;23:2141–51. doi: 10.1016/j.csbj.2024.05.024 (PMC11141151; doi:10.1016/j.csbj.2024.05.024)
Supplement: Supplementary file 1 — Supplementary material [file mmc1.docx]

**SUPPLEMENTARY MATERIALS**

**Addressing docking pose selection with structure-based deep learning: recent advances, challenges and opportunities.**

*Serena Vittorio ^1^, Filippo Lunghini ^2^, Pietro Morerio ^3^, Davide Gadioli ^4^, Sergio Orlandini ^5^, Paulo Miguel Guimarães da Silva ^6^, Jan Martinovic ^6^, Alessandro Pedretti ^1^, Domenico Bonanni ^7^, Alessio Del Bue ^3^, Gianluca Palermo ^4^, Giulio Vistoli ^1*^, Andrea R. Beccari ^2*^*

^1^ Dipartimento di Scienze Farmaceutiche, Università degli Studi di Milano, Via Luigi Mangiagalli, 25, Milano, I-20133, Italy

^2^ EXSCALATE, Dompé Farmaceutici SpA, Via Tommaso de Amicis 95, 80123, Naples, Italy

^3^ Pattern Analysis and Computer Vision, Fondazione Istituto Italiano di Tecnologia, Via Morego, 30, 16163 Genova, Italy

^4^ Dipartimento di Elettronica Informazione e Bioingegneria, Politecnico di Milano, Via Ponzio 34/5, I-20133, Milano, Italy

^5^ SCAI, SuperComputing Applications and Innovation Department, CINECA, Via dei Tizii 6, Rome, 00185, Italy

^6^ IT4Innovations, VSB – Technical University of Ostrava, 17. listopadu 2172/15, 708 00 Ostrava-Poruba, Czech Republic ^7^ Department of Physical and Chemical Sciences, University of L'Aquila, via Vetoio, L'Aquila, 67010, Italy

Corresponding Author: Andrea R. Beccari, EXSCALATE, Dompé Farmaceutici SpA, Via Tommaso de Amicis 95, 80123, Naples, Italy ([andrea.beccari@dompe.com](mailto:andrea.beccari@dompe.com)).

**1. Dataset preparation**

The PDBbind2020 version [1] was downloaded from http://www.pdbbind.org.cn/ website, containing 19433 protein-ligand complexes. Protein and ligand files were pre-processed and standardized by Schrodinger Protein Preparation Wizard [2] and Ligprep [3] utilities using default settings. This step was necessary to solve chemical standardization problems such as atom bumps, missing side chains and loops, wrong valence, missing hydrogens and assigning partial charges.

To collect an overlapping benchmarking dataset, only PDBs which were successfully treated by all scoring functions and pose selection methods were retained. This led to a final dataset of common 12335 complexes.

**2. Molecular docking**

*2.1 Plants*

PLANTS [4] docking was performed by using speed1 as search mode and ChemPLP as scoring function. Ten poses were generated for each ligand. The binding site was defined including the residues within 10 Å from the co-crystallized ligand.

*2.2 Glide*

Glide [5] docking was carried out by using the default glide SP settings.

*2.3 Autodock Vina*

VEGA ZZ [6] was used to convert protein.pdb and ligand.mol2 files into .pdbqt format, which is the standard input for Autodock Vina [7]. Docking was performed with the following settings: binding pocket size = 10 Å, number of generated poses = 10 and exhaustiveness = 8.

**3. Pose selection algorithms**

*3.1 RTM*

RTM [8] is a machine learning-based scoring function for protein-ligand docking. It is characterized by the incorporation of both a customized residue-based graph representation strategy and multiple graph transformer layers designed for learning protein and ligand representations.

Herein, the model was retrained and scored comparably to the models provided by the authors of the paper. The official code can be found at https://github.com/sc8668/RTMScore.

*3.2 DeepDock*

DeepDock [9] is a tool designed for molecular docking and pose selection. It employs deep learning techniques (i.e., Neural Networks (NN), Graph Neural Networks (GNN) and Mixture Density Networks (MDN)) to predict the binding conformation of a given ligand to its target and to rank potential ligand-protein interactions. By leveraging neural networks, DeepDock aims to enhance the accuracy and efficiency of molecular docking simulations, thereby facilitating more precise predictions of ligand binding poses within protein binding sites. This tool is promising for advancing drug discovery and development processes by streamlining the identification of potential therapeutic compounds. The framework is publicly available on GitHub (https://github.com/OptiMaL-PSE-Lab/DeepDock) for usage and further development by the scientific community.

The experimental results presented in this paper were obtained with the following model configurations:

Ligand Model

- Input dimension: 28

- Number of residual layers: 10

- Dropout rate: 0.10

Target Model

- Input dimension: 4

- Number of residual layers: 10

- Dropout rate: 0.10

Overall Model Settings

- Hidden dimension: 64

- Number of Gaussian functions: 10

- Dropout rate: 0.10

- Distance threshold: 7.0

The settings above define the architecture and hyper-parameters of DeepDock model, which combines a ligand model and a target model for molecular docking and pose selection tasks (in our case, only pose selection was used with DeepDock).

The scoring itself was executed adopting the following inputs and respective settings:

- real_mol: Represents a real molecule.

- target_ply: Represents a geometric representation of the target molecule.

- model: The DeepDock model that was previously initialized.

- dist_threshold: Distance threshold set to 3.0.

- seed: Random seed set to 123.

- device: Specifies the device for computation (e.g., CPU or GPU).

The settings above are the inputs of the model and respective settings. They enable the computation of a score that reflects the binding affinity of the real molecule (real_mol) with the target molecule (target_ply) using the default DeepDock model. The seed ensures reproducibility of the results. The computation is performed on the specified hardware: a CPU or a GPU device can be used.

*3.3 GraphBAR*

GraphBAR [10] is graph convolutional neural network for protein-ligand binding affinity prediction. In this work, we have re-implemented the code released by the authors (<https://github.com/jtson82/graphbar>) using PyTorch as Deep Learning library. The Network was trained to predict poses RMSD instead of binding affinity.

*3.4 Dimenet*

DimeNet [11] was originally designed for predicting quantum mechanical properties of molecules.

We adapted and retrained the improved Dimenet++ [12] for the prediction of RMSD between crystal and docked poses. The official code can be found at: <https://github.com/gasteigerjo/dimenet>. As suggested by the authors, we used the Pytorch-geometric implementation (https://github.com/pyg-team/pytorch_geometric/blob/master/torch_geometric/nn/models/dimenet.py).

**References**

[1] Wang R, Fang X, Lu Y, Wang S. The PDBbind Database: Collection of Binding Affinities for Protein−Ligand Complexes with Known Three-Dimensional Structures. *J Med Chem* 2004;47:2977–80. https://doi.org/10.1021/jm030580l.

[2] Madhavi Sastry G, Adzhigirey M, Day T, Annabhimoju R, Sherman W. Protein and ligand preparation: parameters, protocols, and influence on virtual screening enrichments. *J Comput Aided Mol Des* 2013;27:221–34. https://doi.org/10.1007/s10822-013-9644-8.

[3] Schrödinger Release 2023-4: LigPrep, Schrödinger ,LLC, New York, NY, 2023..

[4] Korb O, Stützle T, Exner TE. PLANTS: Application of Ant Colony Optimization to Structure-Based Drug Design, 2006, p. 247–58. https://doi.org/10.1007/11839088_22.

[5] Friesner RA, Banks JL, Murphy RB, Halgren TA, Klicic JJ, Mainz DT, et al. Glide: A New Approach for Rapid, Accurate Docking and Scoring. 1. Method and Assessment of Docking Accuracy. *J Med Chem* 2004;47:1739–49. https://doi.org/10.1021/jm0306430.

[6] Pedretti A, Mazzolari A, Gervasoni S, Fumagalli L, Vistoli G. The VEGA suite of programs: an versatile platform for cheminformatics and drug design projects. *Bioinformatics* 2021;37:1174–5. https://doi.org/10.1093/bioinformatics/btaa774.

[7] Trott O, Olson AJ. AutoDock Vina: Improving the speed and accuracy of docking with a new scoring function, efficient optimization, and multithreading. *J Comput Chem* 2010;31:455–61. https://doi.org/10.1002/jcc.21334.

[8] Shen C, Zhang X, Deng Y, Gao J, Wang D, Xu L, et al. Boosting Protein-Ligand Binding Pose Prediction and Virtual Screening Based on Residue-Atom Distance Likelihood Potential and Graph Transformer. *J Med Chem* 2022;65:10691–706. https://doi.org/10.1021/acs.jmedchem.2c00991.

[9] Méndez-Lucio O, Ahmad M, del Rio-Chanona EA, Wegner JK. DeepDock Paper. *Nat Mach Intell* 2021;3:1033–9.

[10] Son J, Kim D. Development of a graph convolutional neural network model for efficient prediction of protein-ligand binding affinities. *PLoS One* 2021;16:1–13. https://doi.org/10.1371/journal.pone.0249404.

[11] Gasteiger J, Groß J, Günnemann S. Directional Message Passing for Molecular Graphs. 8th Int Conf Learn Represent ICLR 2020 2020:1–13.

[12] Gasteiger J, Giri S, Margraf JT, Günnemann S. Fast and Uncertainty-Aware Directional Message Passing for Non-Equilibrium Molecules 2020.
